# The inappropriate use of antibiotics in upper respiratory tract infections: it is time for action^☆^

**DOI:** 10.1016/j.bjorl.2015.12.003

**Published:** 2016-01-04

**Authors:** Antonio Carlos Campos Pignatari, Monica Menon Myake

**Affiliations:** a Discipline of Infectious Diseases, Universidade Federal de São Paulo (UNIFESP), São Paulo, SP, Brazil; b Núcleo de Otorrinolaringologia do Hospital Sírio Libanês, São Paulo, SP, Brazil

Antibiotics are one of the greatest discoveries in medicine and have significantly decreased mortality and morbidity rates for infectious diseases during the last 75 years. However, large quantities of antibiotics are utilized empirically and inappropriately, particularly for upper respiratory tract infections.

The vast majority of these community-acquired infections is initially caused by viruses, self-limited in their clinical evolution, and have no need of antibiotic treatment. A small percentage is complicated by secondary bacterial infection for which antibiotics could be useful. Several diverse factors can be responsible for this inappropriate use of antibiotics. There is a lack of fast and accurate laboratory tests do differentiate bacterial from viral infections; patients sometimes believe that antibiotics can relieve symptoms such as fever and pain and pressure doctors to prescribe antibiotics when their symptoms do not improve quickly with other medical treatment; doctors practice defensive medicine, antibiotics are available without prescriptions in many countries, and doctors have difficulty identifying patients at high risk of bacterial complications, such as the elderly, immunocompromised and carriers of chronic diseases.

Brazil is the fourth largest global consumer of medicines and 40% of these are antibiotics. Since 2011 Brazilian pharmacies are not allowed to sell antibiotics without a medical prescription and since 2013 all pharmacies have to submit an electronic communication about antibiotics prescriptions to the ANVISA (National Agency for Sanitary Vigilance). In the first year after the implementation of this policy there was a 20% decrease in antibiotics prescriptions, but soon thereafter, the number increased again.

Allergy, collateral effects such as gastrointestinal symptoms and toxicity (hepatic, renal, neurologic, cardiac and teratogenicity) are well described, and can occur with drug usage from the majority of the antibiotics classes. Resistance to antibiotics, sometimes with multiresistant bacteria, is usually seen in nosocomial infections but also occurs in community infections.

Currently there are few new available classes of antibiotics, and it is very important to preserve the commonly used antibiotics particularly the class of B-lactams that is characterized by low toxicity in the majority of patients including neonates, children, pregnant woman and the elderly. When they are indicated the basic criteria for antibiotic usage should be reinforced constantly in the treatment based on international and local guidelines for each category of infection. Collateral effects and toxicity are relevant for the patient as an individual but bacterial resistance is relevant for the entire community.

Another important recent topic is the long-term effects of antibiotics on the human microbiome that may persist throughout an entire life span and is probably associated with chronic inflammatory diseases and even to some types of neoplasia.

All these issues are important and are addressed globally by different institutions and scientific societies, governments, ONGs and private organizations promoting the rational use of antibiotics. Doctors, pharmacists and patients are co-responsible for the success of this endeavor and otorhinolaryngologists must be fully involved. More efforts in education, training and research are warranted, but the effort of each individual is essential at this moment.

## Conflicts of interest

The authors declare no conflicts of interest.
